# Dynamic Active Sites in Bi_5_O_7_I Promoted by Surface Tensile Strain Enable Selective Visible Light CO_2_ Photoreduction

**DOI:** 10.34133/2022/9818792

**Published:** 2022-10-12

**Authors:** Xian Shi, Xing'an Dong, Yanjuan Sun, Shihan Zhang, Fan Dong

**Affiliations:** ^1^Research Center for Environmental and Energy Catalysis, Institute of Fundamental and Frontier Sciences, University of Electronic Science and Technology of China, Chengdu 611731, China; ^2^School of Resources and Environment, University of Electronic Science and Technology of China, Chengdu 611731, China; ^3^Key Laboratory of Microbial Technology for Industrial Pollution Control of Zhejiang Province, College of Environment, Zhejiang University of Technology, Hangzhou 310014, China

## Abstract

Surface defects with abundant localized electrons on bismuth oxyhalide catalysts are proved to have the capability to capture and activate CO_2_. However, bismuth oxyhalide materials are susceptible to photocorrosion, making the surface defects easily deactivated and therefore losing their function as active sites. Construction of deactivation-resistant surface defects on catalyst is essential for stable CO_2_ photoreduction, but is a universal challenge. In this work, the Bi_5_O_7_I nanotubes with surface tensile strain are synthesized, which are favorable for the visible light-induced dynamic I defects generation. The CO_2_ molecules absorbed on I defects are constantly reduced by the incoming photogenerated electrons from I-deficient Bi_5_O_7_I nanotubes and the successive protonation of CO_2_ molecules is thus highly promoted, realizing the selective CO_2_ conversion process via the route of CO_2_-COOH^−^-CO. The efficient and stable photoreduction of CO_2_ into CO with 100% selectivity can be achieved even under visible light (*λ* >420 nm) irradiation benefited from the dynamic I defects as active sites. The results presented herein demonstrate the unique action mechanism of light-induced dynamic defects during CO_2_ photoreduction process and provide a new strategy into rational design of deactivation-resistant catalysts for selective CO_2_ photoreduction.

## 1. Introduction

Photocatalytic conversion of carbon dioxide (CO_2_) with water (H_2_O) into valuable chemical fuels or feedstocks is a promising approach for efficiently relieving the energy issue and carbon emission crisis [1, 2]. Despite the promising prospect, photocatalytic CO_2_ conversion still faces a great challenge in terms of catalytic activity and product selectivity. The low reactivity of CO_2_ in chemical transformations limits the catalytic activity and the multiple side reactions along with the main reaction lower the product selectivity [3–5]. The efficiency of photocatalytic CO_2_ conversion is dependent on the catalytic active sites on the catalyst surface [6–8]. The surface nature and electronic structures of catalytic sites are two versatile knobs for regulating catalytic activity and selectivity, which can be tailored by defect engineering [9].

The introduction of defects into the photocatalysts can accelerate the separation efficiency of electron–hole pairs [10–14]. Additionally, a high product selectivity can be achieved mediated by defects as catalytic active sites via regulating the redox kinetics of CO_2_ photoreduction [2, 11]. However, such synthetically introduced surface defects can be easily occupied or even filled by ambient molecules (O_2_, H_2_O and other molecules) during long-duration CO_2_ photoreduction, leading to the deactivation of defects and the termination of CO_2_ activation [15]. Hence, the construction of photocatalyst surface with continuous supply of defects should be a reliable strategy toward efficient photocatalytic CO_2_ conversion.

Recently, we have explored the feasibility of CO_2_ photoreduction performance improved by light-induced surface Cl dynamic defects [16, 17]. The dynamic evolution of Cl defects excited by light irradiation resulted in the generation of abundant surface defects as active sites for sustainable photocatalysis. Apart from the light field, the external electric field, magnetic field and gas pressure of catalytic systems could also have influence on the dynamic evolution of defect structure [18]. Nevertheless, the effects of surface tensile strain of catalyst, an inherent factor for surface atoms organization, on dynamic defects evolution have not been revealed. The surface tensile strain usually exists when two dimensional (2D) layered photocatalysts are curved, leading to the distortion of surface structure [19]. The surface interatomic distance along the curved direction could be stretched due to the atomic tensile strain [20, 21]. This is favorable for the partial escape of surface atoms or ions to form defects [5], which could furnish an excellent platform for the generation of light-induced dynamic defects on catalyst surface. However, to the best of our knowledge, the dynamic defects promoted by surface tensile strain under external light field together with its effects on CO_2_ photoreduction have not been reported yet.

As a proof-of-concept demonstration, the 2D ultrathin Bi_5_O_7_I is selected as a model system to perform our investigation. Surface tensile strain is engineered by curving the Bi_5_O_7_I to form nanotubes via adjusting the synthesis temperature. As expected, the surface tension of Bi_5_O_7_I nanotubes creates favorable conditions for the migration of iodine ions, forming a certain amount of dynamic iodine defects during CO_2_ photoreduction process under visible light illumination (*λ* >420 nm), which is the essential reason why the Bi_5_O_7_I nanotubes possess CO_2_ photoreduction activity under visible light. The strain effect at the atomistic scale can promote the charge separation of Bi_5_O_7_I nanotubes and the dynamic I defects formed during photocatalysis further accelerate this process. The dynamic defects with abundant localized electrons on catalyst surface continuously import the electrons to the adsorbed CO_2_ molecules to promote the successive protonation process. The reaction pathway of visible-light-driven CO_2_ photoreduction is modulated with the dynamic I defects in ultrathin Bi_5_O_7_I nanotubes. The conversion process of CO_2_-COOH^−^-CO is achieved absolutely, thus highly promoting the selectivity up to 100%. This work clarifies the relationship between dynamic defects and CO_2_ photoreduction activity, and provides new insights into rational design of highly active catalysts toward solar energy conversion and heterogeneous catalysis.

## 2. Results

### 2.1. Structural Features and CO_2_ Photoreduction Properties of Ultrathin Bi_5_O_7_I Nanotubes and Nanoplates

**Figure 1:**
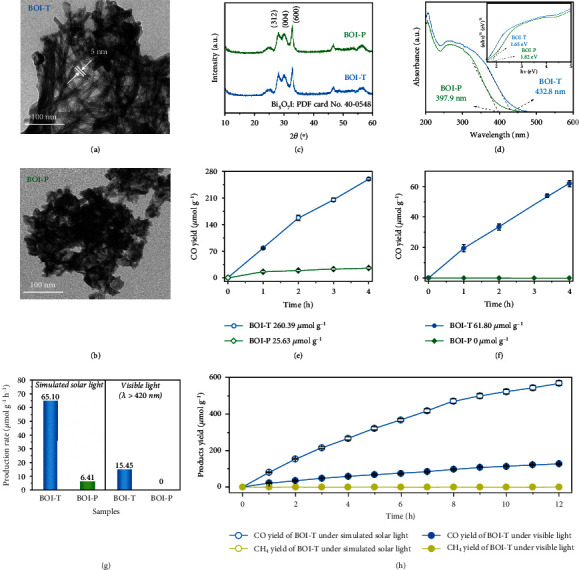
TEM images of BOI-T and BOI-P (a) and (b); XRD patterns (c) and UV-vis DRS of catalysts, and the inset pattern is Tauc plot (d). Amount of CO generation with time under simulated solar light and visible light (e) and (f); CO generation rates under simulated solar light and visible light (g); CO_2_ photoreduction of BOI-T for 12 h under simulated solar light and visible light (h).

Figure S1 illustrated the typical synthesis mechanism of ultrathin Bi_5_O_7_I photocatalysts. The ordered oleylamine-iodine-bismuth (OA-I^−^-Bi^3+^) complexes were formed by the complexation of Bi^3+^, I^−^ and oleylamine (OA) mixture. The addition of water furnished the O atoms for the Bi_5_O_7_I structure formation [15]. The OA-I^−^-Bi^3+^ complexes were gradually hydrolyzed and then self-assembled into different microstructures under different synthesis temperature. At 20°C, a large amount of Bi_5_O_7_I nanoplates were formed, accompanied by a few short ultrafine nanotubes (Figure S2). The resulted products were gradually transformed from nanoplates to short ultrafine nanotubes when synthesis temperature was increased to 40°C (Figure S3). Figure S1 showed that the OA-Bi-I precursor could be fully hydrolyzed at 60°C and the pure long ultrafine nanotubes with a diameter of 5 nm were formed (Figure 1(a)). However, the Bi_5_O_7_I prepared at 80°C was completely transformed into the morphology of nanoplates (Figure 1(b)). Synthesis temperature of 60°C could lead to the complete hydrolysis of the OA-Bi-I complexes, while lower or higher temperature would hinder the hydrolysis process, so that only short and aggregated nanotubes or nanoplates could be produced. To explore the effect of surface tensile strain on light-induced defects and photocatalysis reaction, a comparative study was carried out using pure long ultrafine Bi_5_O_7_I nanotubes (BOI-T) and ultrathin Bi_5_O_7_I nanoplates (BOI-P) as models.

As shown in Figure 1(c), all the diffraction peaks corresponding to BOI-T and BOI-P were indexed to standard monoclinic phase Bi_5_O_7_I (PDF card No. 40-0548) [22]. The XRD patterns of Bi_5_O_7_I prepared at 20 and 40°C were shown in Figure S4. The extended light absorption of BOI-T compared with BOI-P was determined from UV-vis DRS in Figure 1(d). The bandgaps of the BOI-T and BOI-P were estimated to be 1.65 and 1.82 eV, respectively (right inset of Figure 1(d)). X-ray photoelectron spectroscopy (XPS) was used to examine the valence state of elements. The high-resolution XPS spectra of Bi 4f showed two main peaks attributing to 4f_5/2_ at 164.2 eV and 4f_7/2_ at 158.9 eV (Figure S5), suggesting the typical Bi^3+^ oxidation state [23]. As shown in Figure S6, besides the main Bi-O peak at 529.9 eV, another peak appeared at 532.2 eV was arisen from the surface hydroxyl groups [22, 24]. The peaks located at 618.7 and 630.2 eV were attributed to I 3d_5/2_ and I 3d_3/2_ of I^−^ in Bi_5_O_7_I (Figure S7) [22].

The BOI-T indeed possessed apparent photocatalytic activity toward CO_2_ conversion to CO under simulated solar light and visible light irradiation. As shown in Figure S8 and S9, no reduction product was detected without a photocatalyst or under an Ar atmosphere. Figure 1(e) showed that the CO yield on BOI-T was increased to 260.39 *μ*mol g^−1^ after 4 h of simulated solar light irradiation while the CO yield amount of BOI-P was just 25.63 *μ*mol g^−1^ under the same conditions. After 4 h irradiation of visible light (*λ* >420 nm), nearly no CO was generated by BOI-P, suggesting that it could not utilize visible light to reduce CO_2_. In contrast, a CO yield of 61.80 *μ*mol g^−1^ could be observed over BOI-T under visible light. The CO yield rates under simulated solar light and visible light were shown in Figure 1(g). Notably, no CH_4_ or H_2_ was produced (Figure S10 and S11) and only CO was detected during the reduction process suggesting a 100% product selectivity, which was further confirmed by the continuous CO_2_ photoreduction experiments for 12 h (Figure 1(h) and S12). The ^13^CO_2_ isotopic labeling experiments were performed with BOI-T. A peak at m/z =29 corresponding to ^13^CO was observed from the mass spectroscopy (Figure S13), indicating that the produced CO was originated from the CO_2_ photoreduction. Simultaneously, the peaks corresponding to ^13^CH_4_ were not observed during the test, this further confirmed the 100% CO selectivity. As mentioned above, the surface tensile strain existed in Bi_5_O_7_I nanotubes, and this might be the reason why the Bi_5_O_7_I with different morphologies exhibited obvious differences in CO_2_ photoreduction performance. Thus, the unprecedented high CO yield over BOI-T compared with BOI-P, especially under visible light might benefit from the high surface tensile strain on BOI-T.

### 2.2. Surface Tensile Strain of Ultrathin Bi_5_O_7_I Nanotubes

**Figure 2:**
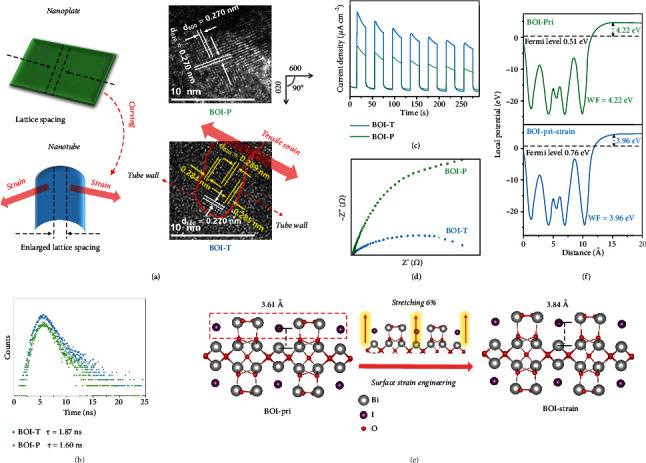
Surface tensile strain formation process and related enlarged HRTEM images (a); Time-resolved transient PL decay (b); Transient photocurrent responses (c); EIS plots (d); Structural models of pristine BOI and BOI under strain effect performed by DFT. The BOI under strain effect model was obtained by stretching the surface atoms upward by 6% (e); Work functions of pristine BOI and BOI under strain effect (f).

Surface tensile strain engineered by curing the nanoplate into nanotube was illustrated in Figure 2(a). The HRTEM images showed more intuitive evidence. For BOI-P, the lattice fringes of 0.27 nm were observed with the angle of 90°, corresponding to the (600) and (020) crystal planes of Bi_5_O_7_I [25]. Under the effect of surface tensile strain, the lattice spacing of (600) crystal plane perpendicular to the tube wall of BOI-T was still 0.270 nm, while the lattice spacing of (020) crystal plane parallel to the tube wall increased to ∼0.286 nm, providing an intuitive evidence of an anisotropic lattice tensile strain (~6%, [0.286 - 0.270]/0.286 ≈ 6%) along the Bi_5_O_7_I nanotubes [26].

The enhanced carrier separation enhanced by the inherent surface tensile strain of BOI-T had been proved. The protracted photoluminescence (PL) lifetime of BOI-T relative to BOI-P (1.87 vs 1.60 ns) could be observed in Figure 2(b), indicating that the nanotubes possessed higher charge separation efficiency.^15^ In Figure 2(c), BOI-T showed an enhanced photocurrent intensity in comparison with BOI-P, suggesting a better charge transfer ability within the nanotubes. Meanwhile, BOI-T also displayed a much smaller semicircle radius of EIS Nyquist plots, indicating its lower charge transfer resistance (Figure 2(d)) [27, 28]. Additionally, the lower EPR signal of BOI-T in Figure S14 signified more photogenerated electrons consumption due to the enhanced charge separation. Figure 2(e) showed the DFT calculation results performed with the structural models of pristine BOI (BOI-pri) and surface tensile strain tuned BOI (BOI-strain). The BOI-strain was obtained by stretching the surface atoms of BOI-pri upward by 6% according to the experimental results in Figure 2(a). In Figure 2(f), the electronic work functions (WFs) of BOI-pri and BOI-strain were calculated as 4.22 and 3.96 eV, respectively. The smaller WF value of BOI-strain suggested a lower barrier for the transferring of photo-generated electrons and a strengthen electronic transmission capability [29], which was consistent with the charge separation enhanced by the inherent surface tensile strain of nanotubes from experimental results.

### 2.3. Formation of Light-Induced Reversible I Defects Promoted by Surface Tensile Strain of Ultrathin Bi_5_O_7_I Nanotubes

**Figure 3:**
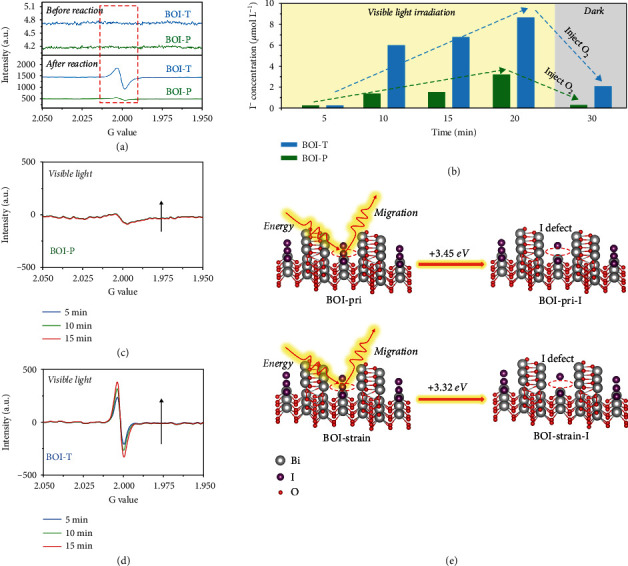
EPR signals of defects in BOI-P and BOI-T before and after 12 h CO_2_ photoreduction under visible light illumination (a); Variation trend of free I^−^ in solution detected by ICP under visible light illumination (b); EPR signals for defects of BOI-P and BOI-T under 15 min continuous visible light illumination, the signal was recorded at a 5 min interval (c, d); Energy of I defect formation of BOI-pri and BOI-strain performed by DFT (e).

The surface interatomic distance along the curved direction will inevitably enable the escape of partial surface atoms or ions to form defects [5]. Simultaneously, the enlarged surface areas of BOI-T (Figure S15) allowed more exposed atoms on its surface, which would be favorable for light-induced defects generation [15]. This was ascertained by XRD change in Figure S16 and S17. In comparison to pristine BOI-T, the as-used catalyst gradually exhibited a series of new diffraction peaks at 24.51, 27.46, 30.12, 32.61, 37.97, 39.69 and 48.52° corresponding to Bi_2_O_3_ (PDF card no. 45-1344) after more than 4 h visible light irradiation [6]. The small amount of generated Bi_2_O_3_ should be attributed to the light-induced migration of I^−^ from the crystal structure, which would result in the formation of I defects. The EPR signal at around *g* =2.000 was a typical feature of anion defects, which can be attributed to the in-situ formed I defects induced visible light. (Figure 3(a)). Analogously, the extremely tiny change on XRD patterns of BOI-P indicated fewer defects formation, which was also affirmed by EPR test (Figure 3(a)).

In order to track and confirm the light-induced dynamic I defects evolution, the experiments were carried out in the liquid phase under pure N_2_ atmosphere to directly observe the variation in the amount of I^−^ in solution by an inductive coupled plasma emission spectrometer (ICP). As shown in Figure 3(b), compared with BOI-P, the BOI-T could make a rapid response to the release of I^−^, which was conducive to the light-induced I defects generation. As the color of BOI-T changed from white to black under visible light irradiation (Figure S18), the amount of free I^−^ in solution gradually increased, suggesting that the I ions migrated from the crystal structure of BOI-T to surface. A certain amount of visible-light-induced dynamic I defects were in-situ formed during this process owing to the surface tensile strain. Nevertheless, the slight change of I^−^ in solution over BOI-P under the same conditions implied that visible-light-induced I defects were difficult to be formed on its surface. This should be the reason why BOI-P lost its ability for CO_2_ reduction under visible light. Meanwhile, we found that when the O_2_ was bubbled into the black sample under dark condition, the concentration of free I^−^ in solution decreased sharply (Figure 3(b)) and the black sample returned to white. The XRD patterns of the black sample after 20 min of visible light illumination (Figure S19) and the recovered sample (Figure S20) confirmed that the generation of Bi_2_O_3_ could be regarded as a sign of light-induced I defects formation and the BOI-T was recoverable by a facile method.

To further intuitively confirm the dynamic I defects formation, the solid-state EPR signals were recorded under continuous light irradiation. The increasing signals at around *g* =2.000 with prolonged visible light irradiation time suggested that the light-induced dynamic defects were constantly formed on BOI-T (Figure 3(d)). The more obvious EPR signal change of BOI-T further indicated that BOI-T was more prone to generate dynamic I defects on its surface (Figures 3(c) and 3(d)). We also repeated the same experiments under simulated solar light irradiation, and the same conclusion was obtained. The dynamic I defects were easier to form on BOI-T surface than BOI-P (Figure S21, S22, S23 and S24). The stronger EPR signal detected under simulated solar light than that under visible light could account for the higher solar light driven CO_2_ reduction activity of BOI in Figure 1. The contribution of dynamic I defects to CO_2_ photoreduction was fully emphasized. In addition, it could be observed from the ICP and EPR experimental results that the generation ability of light-induced defects decreases slightly with the prolonged illumination time, this might be the reason that the CO_2_ photoreduction performance of BOI-T was slightly reduced in long-period experiments (Figure 1(h)). From DFT calculation in Figure 3(e), the energy required for I^−^ migrated from BOI-strain was obviously lower than that of BOI-pri, indicating the surface tensile strain was the key factor for I defects formation.

### 2.4. The Effect of Light-Induced I Defects on Selective CO_2_ Photoreduction

The discolored BOI-T after visible-light irradiation treatment in liquid phase was freeze dried and used to explore the effect of I defects toward photocatalysis. The formation of I defects will change the binding environment of the O atoms within BOI-T. As shown in Figure S25, the light-treated BOI-T possessed three peaks in its O 1 s XPS spectrum, while the pristine BOI-T only had two peaks (Figure S6). The new shoulder peak appeared at 530.7 eV could be ascribed to the I defects formation. Due to the release of I atoms from BOI-T, the concentration of the positive charges on Bi atom in defective BOI-T at the edge part was decreased. As a result, the electron screening effect for the related bridging hydroxyls was decreased [6, 30]. The peak appeared at 531.9 eV for the light-treated BOI-T was assigned the open metal sites caused by I^−^ migration, which resulted in a strong interaction with the ambient hydroxyl groups. The EPR test for defect trapping shown in Figure S26 indicated that a certain amount of I defects were indeed formed on the light-treated BOI-T.

**Figure 4:**
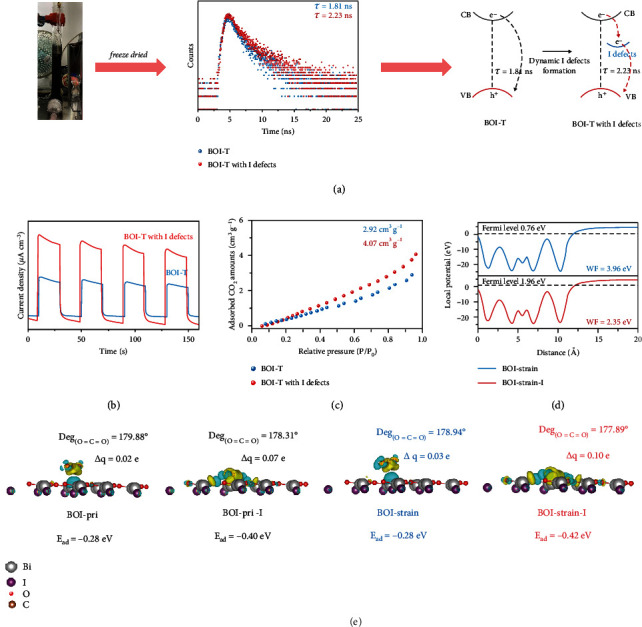
Time-resolved transient PL decay of BOI-T and BOI-T with I defects (a); Transient photocurrent responses of BOI-T and BOI-T with I defects (b); CO_2_ adsorption isotherms of BOI-T and BOI-T with I defects at 25°C (c); Work functions of BOI-strain and BOI-strain-I (d). Structural models of CO_2_ adsorbed on BOI-pri, BOI-pri-I, BOI-strain and BOI-strain-I. E_ad_ represented the adsorption energy, *Δ*q represented the Bader values of adsorbed CO_2_, the negative values meant that CO_2_ gained electrons, the isosurfaces were set to 0.0034 e Å^−1^, and deg_(O=C=O)_ represented the angle of O=C=O bond (e).

The increased decay time from 1.81 (untreated BOI-T) to 2.23 ns (discoloured BOI-T) indicated that the photogenerated electrons could be first trapped by I defects and then recombined with holes, leading to a longer average decay time as shown in Figure 4(a). This also indicated an improved charge separation efficiency, which was consistent with the enhanced photocurrent intensity as shown in Figure 4(b). The enhanced charge separation also resulted in a lower EPR signal for electron trapping (Figure S27). Figure S28 showed the UV-vis DRS spectra of BOI-T before and after the generation of visible-light-induced I defects. Generally, the presence of defects in bismuth oxyhalide-based materials is supposed to induce indirect sub-band excitation, which can alter their optical properties [31]. As expected, a red shifting of light absorption was observed over light-treated BOI-T, and its bandgap was slightly increased from 1.65 to 1.97 eV (Figure S29). Normally, photocatalysts will be endowed with extra surface features after surface defects generation, such as the enhanced adsorption of reaction substrates [31]. In Figure 4(c), the generation of I defects enabled the light-treated BOI-T to show 1.4 times enhancement for CO_2_ adsorption in comparison with untreated BOI-T at 25°C (4.07 *vs* 2.92 cm^3^ g^−1^), which could be ascribed to more adsorption sites for CO_2_ provided on I defects.

The role of light-induced I defects was revealed by DFT calculations. In Figure 4(d), the WFs of BOI-strain and BOI-strain-I were calculated as 3.96 and 2.35 eV, respectively. Similarly, this result suggested an enhanced electronic transmission capacity of BOI-strain-I. The adsorption energy of CO_2_ adsorbed on BOI-strain-I (-0.42 eV) was more negative than those on BOI-pri-I (-0.40 eV), BOI-strain (-0.28 eV) and BOI-pri (-0.28 eV) (Figure 4(e)), suggesting the enhanced CO_2_ adsorption ability on I defects, which was in agreement with the result in Figure 4(c). Bader analysis demonstrated that the I defects on BOI-strain-I could efficiently promote the charge exchange for adsorbed CO_2_ molecules. The CO_2_ adsorbed on BOI-strain-I gained more electrons (*Δq* = -0.10 e) than those adsorbed on other models. The maximum bending of O=C=O bond (177.89°) of CO_2_ molecules adsorbed on BOI-strain-I suggested that CO_2_ molecules were more easily activated under the effect of I defects. We could thus conclude from these results that the dynamic I defects formed on surface tensile strain tuned Bi_5_O_7_I could further accelerate charge separation and act as adsorption/active sites to boost the CO_2_ photoreduction process.

**Figure 5:**
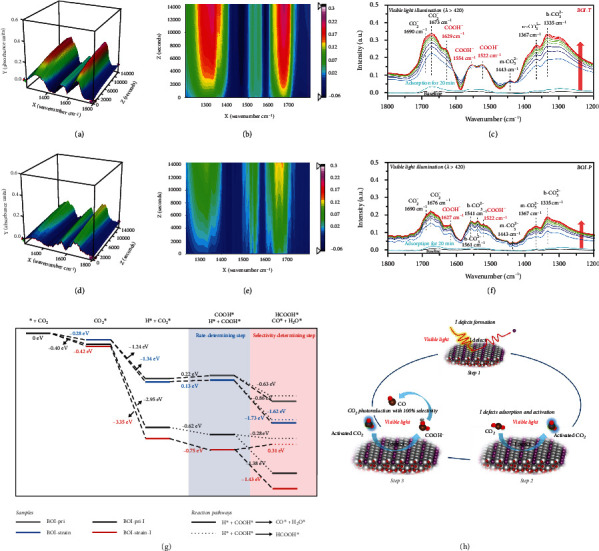
3D FT-IR spectra evolution process of the CO_2_ photoreduction process over BOI-T and BOI-P during visible light irradiation for 180 cycles (0-14000 s) (a, d); 3D spectra evolution for BOI-T and BOI-P (b, e); In situ FT-IR spectra of CO_2_ photoreduction process over BOI-T and BOI-P recorded during visible light irradiation cycles of 1, 4, 10, 20, 40, 80, 120, 140, 160 and 180 (from bottom to top) (c, f); Reaction pathways for CO_2_ photoreduction over BOI-pri, BOI-pri-I, BOI-strain and BOI-strain-I based on DFT calculation, ‘∗' represented the adsorption site on the substrate (g); The CO_2_ photoreduction mechanism promoted by light-induced I defects on surface strain tensile tuned BOI (h).

The rapid scan in-situ Fourier-transform infrared (FT-IR) spectroscopy was used to track the intermediate products of CO_2_ photoreduction over BOI-T and BOI-P. The adsorption processes on BOI-T and BOI-P were measured for 20 min to ensure that the adsorption of CO_2_ and H_2_O mixture reached equilibrium in the dark. The stronger intensity of IR curve after 20 min adsorption process of BOI-T than that of BOI-P was ascribed to the stronger CO_2_ adsorption capacity of BOI-T. The CO_2_ photoreduction under visible light illumination were shown in Figures 5(a)–5(f). For BOI-T, the IR peaks appeared at 1690 and 1673 cm^−1^ were assigned to the activated CO_2_ (CO_2_^−^). The signals corresponding to m-CO_3_^2-^ (1443 and 1367 cm^−1^) and b-CO_3_^2-^ (1335 cm^−1^) ascribed to the asymmetric O − C − O stretch of monodentate and bidentate carbonate groups [32–36]. The IR peaks of COOH^−^ increased with illumination time were found at 1629, 1554 and 1522 cm^−1^. The continuous generation of COOH^−^ is regarded as a critical and typical step in the CO_2_-to-CO conversion [26, 37–40]. For BOI-P, in addition to the IR peaks corresponding to CO_2_^−^ (1690 and 1673 cm^−1^), m-CO_3_^2-^ (1443 and 1367 cm^−1^) and b-CO_3_^2-^ (1335 cm^−1^), the peaks at 1561 and 1541 cm^−1^ indexed to b-CO_3_^2-^ suggested that a considerable part of CO_2_ molecules were just adsorbed on BOI-P under light irradiation [39, 40], resulting in the less COOH^−^ production. This was also further confirmed by the tiny change of IR peaks appeared at 1627 and 1522 cm^−1^. The differences of in-situ FT-IR results could be attributed to the fact that BOI-T generated I defects under visible light irradiation. These light-induced I defects with abundant localized electrons provided a favorable electron-rich environment for the transition from adsorbed CO_2_ to COOH^−^. This was further confirmed by more obvious changes of IR curves shown in Figure S30 and S31 due to more defects formation under simulated solar light illumination.

DFT simulations were performed to explore the mechanism of I-defect-mediated catalytic selectivity toward CO production. The CO_2_ molecules were initially adsorbed on the surface of photocatalysts, and hydrogen ions dissociated from H_2_O molecules and participated in the reaction. The reaction energy profile for the photocatalytic CO_2_-to-CO process with the lowest-energy pathways on the surface of BOI-pri, BOI-strain, BOI-pri-I and BOI-strain-I were calculated, as shown in Figure 5(e). And the corresponding structural models of each simulated reaction step were shown in Figure S32, S33, S34 and S35. The crucial rate-determining step for CO production is the hydrogenation of CO_2_^∗^ to COOH^∗^. For the models without defect, the energy barriers of this step were+0.22 and+0.13 eV for BOI-pri and for BOI-strain, respectively, suggesting an endothermic process. The slightly lower energy barrier of BOI-strain compared with BOI-pri was due to the slightly increased electron transfer (0.03 e *vs* 0.02 e, Figure 4(d)) from catalyst surface to CO_2_ molecule benefited from the surface tensile strain. While after the I defects were formed, the energy barrier of BOI-pri-I and BOI-strain-I were -0.75 and -0.62 eV, indicating that the existence of I defect enabled the CO_2_ conversion to COOH^−^ spontaneously. This was because that CO_2_^∗^ absorbed on I defects were constantly reduced by more incoming photogenerated electrons transferred from BOI-strain-I and the successive protonation process was promoted [41], which was in consistent with the bader analysis (Figure 4(d)).

Subsequently, the produced COOH^∗^ intermediates further couple with a proton-electron pair to generate CO and H_2_O or HCOOH, which is regarded as a selectivity-determining step. The energy barriers of CO and H_2_O or HCOOH generation of this step were similar for both BOI-pri and BOI-strain, indicating an undesirable product selectivity. While for BOI-pri-I and BOI-strain-I, the energy barrier of COOH^∗^-CO^∗^ was more negative than that of COOH^∗^-HCOOH^∗^. This means that the dynamic I defects can modulate the pathway of CO_2_ photoreduction, allowing a high CO catalytic selectivity. It should be especially emphasized that the energy barrier of COOH^∗^-CO^∗^ transition on BOI-strain-I was -1.43 eV, while the energy barrier of COOH^∗^-HCOOH transition was +0.31 eV. This result further demonstrated that CO_2_ can be spontaneously and completely reduced to CO, which could provide the sufficient evidence for the 100% CO catalytic selectivity mediated by I defects engineered by surface tensile strain (Figure 1(h)).

Based on the above discussions, we conclude that the visible-light-driven CO_2_ reduction on the surface tensile strain tuned Bi_5_O_7_I can be divided into three main steps as shown in Figure 5(f): (i) part of the I atoms were escaped from the surface of Bi_5_O_7_I nanotubes under visible light irradiation, and sufficient dynamic I defects are constantly formed on its surface; (ii) CO_2_ molecules are chemisorbed and then activated on the I defects; (iii) the photoexcited electrons are injected into the activated CO_2_, which can be reduced to CO with 100% selectivity.

## 3. Discussion

We utilized the surface tensile strain to promote the dynamic active sites formation and boost the CO_2_ photoreduction activity of Bi_5_O_7_I. During the CO_2_ photoreduction process, the dynamic I defects were formed on the Bi_5_O_7_I nanotubes because of the surface tensile strain for atoms exposure and accelerated the charge separation as active sites, leading to a selective CO production (15.45 *μ*mol g^−1^ h^−1^) under visible light irradiation (*λ* >420 nm). On the basis of in-situ FT-IR and DFT calculation results, it was revealed that the dynamic I defects could modulate the reaction pathway of CO_2_ photoreduction, thus achieving a 100% CO_2_-CO selectivity. The dynamic defects evolution and their function mechanisms in photocatalysis could shed new light on the rational design of defective catalysts with high efficiency for superior solar energy conversion.

## 4. Materials and Methods

### 4.1. Sample Preparation

200 mg of Bi(NO_3_)_3_·5H_2_O was firstly dissolved in 20 mL oleylamine under continuous stirring. The resulted solutions were heated to 20, 40, 60, and 80°C, respectively. 20 mL of deionized water containing 50 mg of KI was then added dropwise. The resulted solutions were heated for 48 h under continuous stirring. After reaction, the resulting solutions were separated by centrifugation at 10000 rpm. The obtained photocatalysts were washed with deionized water and ethanol for several times and then vacuum-dried at 80°C for 24 h.

### 4.2. CO_2_ Photoreduction

The CO_2_ photoreduction were carried out in a Labsolar 6A closed circulation system, and a 300-W xenon lamp coupled with an AM 1.5G filter or visible light filter (420 nm cut) was used as the light source. The experimental details were demonstrated in the Supporting Information.

### 4.3. ICP Measurement of Light-Induced I^−^ Migration

20 mg of Bi_5_O_7_I photocatalyst was used in this part under the irradiation of a 300-W xenon lamp (the xenon lamp was coupled with an AM 1.5G filter or visible light filter (420 nm cut)). The concentration of I^−^ formed during the experiment was measured by ICP. The experimental details were shown in the Supporting Information.

### 4.4. Rapid Scan in-Situ FTIR Spectroscopy

The rapid scan in-situ FTIR spectroscopy was carried out on a Nicolet iS50 FTIR spectrometer equipped with a tailor-made reactor and liquid-nitrogen-cooled HgCdTe detector. The experimental details were demonstrated in the Supporting Information.

## Data Availability

All data needed of this study are available in the article and its Supplementary Information files.

## Additional Points

*General*. Thank others for any contributions, whether it be direct technical help or indirect assistance.

## Supplementary Materials

Supplementary Materials The Schematic illustration of the preparation process; HRTEM images and XRD patterns of BOI-20 and BOI-40; XPS survey spectra for Bi 4f, O 1 s and I 3d of BOI-T and BOI-P; CO_2_ photoreduction without photocatalysts; CO_2_ photoreduction in Ar atmosphere; CH_4_ and H_2_ yield during CO_2_ photoreduction; ^13^CO_2_ isotopic labeling experiments; EPR experimental results; BET and N_2_ adsorption-desorption isotherms; XRD of BOI-T and BOI-P after CO_2_ photoreduction; XRD patterns of BOI-T after illumination and recovered BOI-T; ICP experimental results; XPS spectra for O 1 s of discoloured BOI-T; UV-vis DRS experimental results; In-situ FT-IR results; The corresponding structure models at each reaction step; Tables of comparison of the CO_2_ photoreduction activity to yield CO with other catalysts, and Figures S1-S35 and Table S1-S2 are incorporated in the supplementary material.
