# Supplementary material for: Dynamic Active Sites in Bi5O7I Promoted by Surface Tensile Strain Enable Selective Visible Light CO2 Photoreduction
Source: Research (Wash D C). 2022 Oct 12;2022:9818792. doi: 10.34133/2022/9818792 (PMC9590270; doi:10.34133/2022/9818792)
Supplement: Supplementary Materials — The Schematic illustration of the preparation process; HRTEM images and XRD patterns of BOI-20 and BOI-40; XPS survey spectra for Bi 4f, O 1 s and I 3d of BOI-T and BOI-P; CO2 photoreduction without photocatalysts; CO2 photoreduction in Ar atmosphere; CH4 and H2 yield during CO2 photoreduction; 13CO2 isotopic labeling experiments; EPR experimental results; BET and N2 adsorption-desorption isotherms; XRD of BOI-T and BOI-P after CO2 photoreduction; XRD patterns of BOI-T after illumination and recovered BOI-T; ICP experimental results; XPS spectra for O 1 s of discoloured BOI-T; UV-vis DRS experimental results; In-situ FT-IR results; The corresponding structure models at each reaction step; Tables of comparison of the CO2 photoreduction activity to yield CO with other catalysts, and Figures S1-S35 and Table S1-S2 are incorporated in the supplementary material. [file 9818792.f1.docx]

**Supplemental Material**

**Dynamic active sites in Bi_5_O_7_I promoted by surface tensile strain enable selective visible light CO_2_ photoreduction**

Xian Shi^1^, Xing’an Dong^1^, Yanjuan Sun^2^, Shihan Zhang^3^, Fan Dong^1*^

1 Research Center for Environmental and Energy Catalysis, Institute of Fundamental and Frontier Sciences, University of Electronic Science and Technology of China, Chengdu 611731, China.

2 School of Resources and Environment, University of Electronic Science and Technology of China, Chengdu 611731, China.

3 Key Laboratory of Microbial Technology for Industrial Pollution Control of Zhejiang Province, College of Environment, Zhejiang University of Technology, Hangzhou 310014, China.

Correspondence should be addressed to Fan Dong; dfctbu@126.com, [dongfan@uestc.edu.cn](mailto:dongfan@uestc.edu.cn)

**Characterizations**

XRD measurements were carried out at room temperature (Cu-Kα radiation, D/Max RA, Rigaku Corp., Japan). HRTEM images were obtained using a JEM-2100F field emission electron microscope (Japan Electronics Co., Ltd, Japan). XPS measurements were performed on a Thermo Scientific ESCALAB 250Xi X-ray photoelectron spectrometer (Al-Kα, 150 W, reference C 1s = 284.8 eV, Thermo Fisher Scientific, U. S. A). The optical absorption properties were measured by UV-visible DRS (UV-2450, Shimadzu, Japan). The PL spectrum (HITACHI F-7000, Hitachi, Japan) was used to analyse the photocharge separation efficiency. BET was measured with a BSD-PS2 (Beishide Instrument Co., Ltd., China) fully automatic specific surface area and pore distribution instrument. The electrons and I vacancies generation of solid Bi_5_O_7_I powders were determined using a JES-FA200 EPR spectrometer (Japan Electronics Co., Ltd, Japan).

**CO_2_ photoreduction**

The CO_2_ photoreduction performances were carried out in a reactor under a 300-W xenon lamp coupled with an AM 1.5G filter/visible light filter (420 nm cut) (PLS-SXE300UV, Beijing Perfectlight Technology Co., Ltd., China), the gas phase products were analysed with a Labsolar 6A closed circulation system (Beijing Perfectlight Technology Co., Ltd., China). Firstly, ten milligrams of photocatalyst dispersed in distilled water was dried on the surface of a microporous membrane. Subsequently, the pure CO_2_ was injected into the reactor at a constant pressure of about 1 atm after the reaction system was evacuated for several times. The reaction temperature was kept at 298 K using a circulation cooling system (DC-0506, Shanghai Sunny Hengping Scientific Instrument Co., Ltd., China). A GC2002 gas chromatograph (Shanghai Kechuang Chromatography Instrument Co., Ltd., China) was used to analysed the products qualitatively. The CO selectivity was calculated according to the required electrons for CO_2_ reduction as follows:

CO selectivity (%) = [2*φ*(CO)] / [2*φ*(CO) + 8*φ*(CH_4_) + 2*φ*(H_2_)] × 100%.

where *φ*(CO), *φ*(CH_4_)*,* and *φ*(H_2_) represent the yield rate of the products, respectively.

**Evaluation of light-induced I^-^ migration**

Twenty milligrams of Bi_5_O_7_I was dispersed in 60 mL of distilled water in a quartz reactor, which was equipped with an air-cooling system to eliminate the influence of temperature. High-purity N_2_ was bubbled into the suspension at a flow rate of 60 mL min^−1^ for 60 min in the dark. Before turning on the light, the photocatalyst was fully dispersed under continuous stirring for 10 min. The reactor was then illuminated using a 300-W xenon lamp coupled with an AM 1.5G filter/visible light filter (420 nm cut). 0.5 mL of the reaction solution was collected using a syringe at regular intervals and immediately centrifuged to remove the photocatalyst. The concentration of I^-^ formed during the reaction in the aliquot was determined by ICP (iCAP 7000 SERIES, Thermo Scientific, USA). After one illumination cycle, the xenon light was turned off, and O_2_ was bubbled into the suspension at a flow rate of 60 mL min^−1^ for 30 min to regenerate the sample.

**DFT calculations**

DFT calculations were performed by employing the VASP5.4 code, using the generalised gradient approximation with the Perdew–Burke–Ernzerhof exchange-correlation functional. A plane-wave basis set with a cut-off energy of 400 eV within the framework of the projector-augmented wave method was carried out, along with a 3 × 3 × 1 Monkhorst-Pack grid for the Brillouin zone. The Gaussian smearing width was set to 0.2 eV. The van der Waals correction was described by the D2 method of Grimme. All atoms were allowed to relax and converge to 0.01 eV Å^−1^ for all systems. A 156-atom 2 × 3 × 1 supercell of Bi_5_O_7_I (BOI-pri, including 60 Bi, 84 I, and 12 O atoms with a I atom as the adsorption site) was constructed. A I defect was then introduced into BOI (BOI-pri-I, including 60 Bi, 83 I, and 12 O atoms with the I defect as the adsorption site). The adsorption energy (ΔE_ads_) was defined as follows:

Δ*E*_ad_ ＝ *E*_tot_ − (*E*_s_ + *E*_m_),

where *E*_tot_, *E*_s_, and *E*_m_ are the total energies of the adsorption complex, material, and CO_2_ molecule, respectively.

The Gibbs free energy (Δ*G*) for all CO_2_ reduction reactions was defined as follows:

Δ*G* =Δ*E* + ΔZPE – *T*Δ*S*,

where Δ*E*, ΔZPE, *T*, and Δ*S* are the DFT energy of the adsorption complex, zero-point energy difference, reaction temperature, and entropy difference between the adsorbed complex and the gas phase, respectively.

The construction of model of BOI under surface tensile strain is described in manuscript, and the I defective model is constructed used above method.

**Rapid scan in-situ FTIR spectroscopy**

The rapid scan in-situ FTIR spectroscopy was carried out on a Nicolet iS50 FTIR spectrometer (Thermo Fisher Scientific, USA) equipped with a tailor-made reactor and liquid-nitrogen-cooled HgCdTe detector. Before the adsorption/desorption process, the loaded samples were purged with Ar (50 mL min^−1^) for 1-h at 120 °C to remove all impurities. The background spectrum was then collected after the chamber temperature was lowered to room temperature. Next, the reaction gas mixture (25 mL min^−1^ of Ar and 5 mL min^−1^ of CO_2_ with a trace of H_2_O vapour) was introduced into the reactor, and the variation of the FTIR spectra were recorded to monitor the dynamic adsorption process. After reaching sorption equilibrium (30 min), the background spectrum was collected again. A 300-W xenon lamp equipped with an AM1.5 ﬁlter or a visible light filter (420 nm cut) was employed as the light source, and a chopped irradiation program with 180 cycles of 20 s irradiation and 6 s dark was performed by periodically intercepting the laser beam with a mechanical shutter (Vincent Associates, model Uniblitz). The shutter was controlled by a BNC pulse/delay generator model 565 and synchronized with the data acquisition in an FT-IR spectrometer. The operando IR spectra with a spectral resolution of 4 cm^-1^ and scanning velocity of 160 kHz were collected in each 6 s dark period of the chopped irradiation program.

**Supplementary Figures**


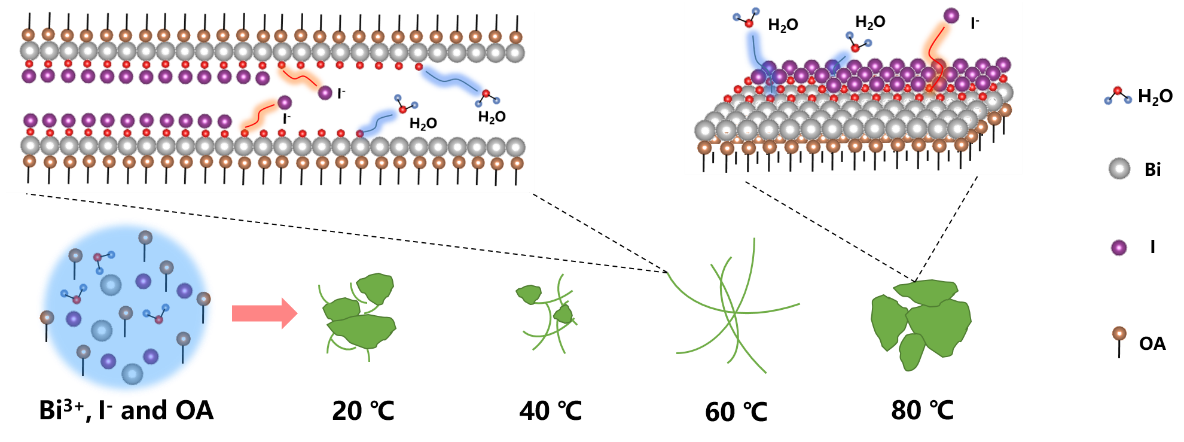


**Figure S1** Schematic illustration of the preparation process of ultrathin BOI under different synthesis temperature.


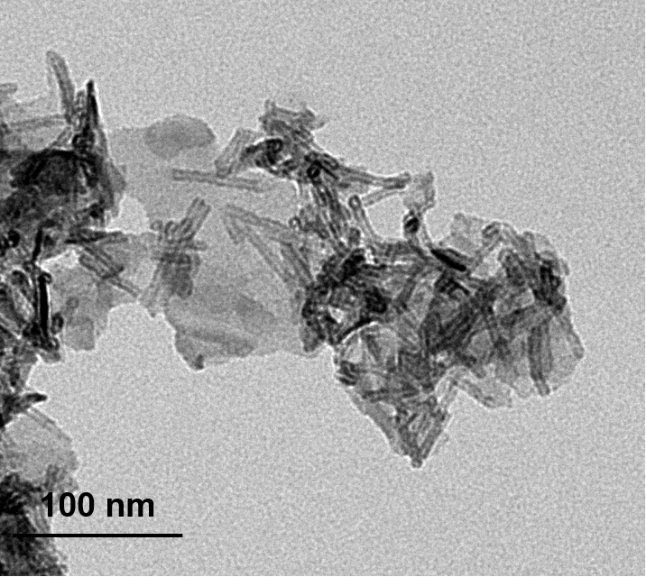


**Figure S2** HRTEM image of Bi_5_O_7_I prepared at 20 ℃.


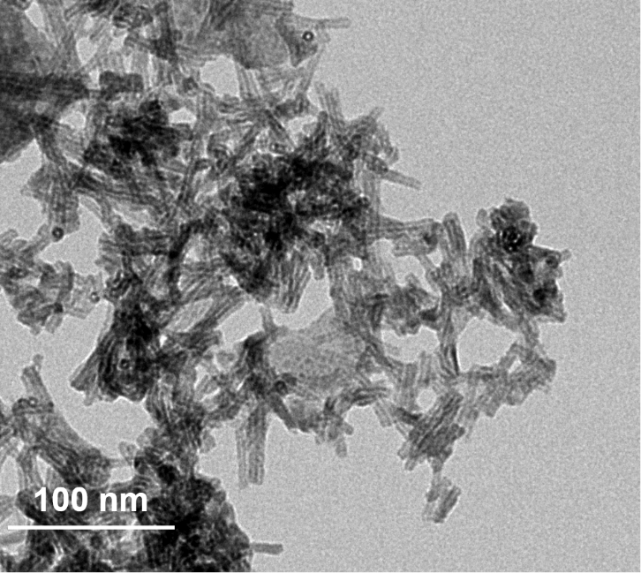


**Figure S3** HRTEM image of Bi_5_O_7_I prepared at 40 ℃.





**Figure S4** XRD patterns of Bi_5_O_7_I prepared at 20 and 40 ℃. BOI-20 (light grey line) represents Bi_5_O_7_I prepared at 20 ℃, and BOI-40 (dark grey line) represents Bi_5_O_7_I prepared at 40 ℃.





**Figure S5** XPS spectra for Bi 4f of BOI-T and BOI-P, green line represents BOI-P, blue line represents BOI-T.





**Figure S6** XPS spectra for O 1s of BOI-T and BOI-P, green line represents BOI-P, blue line represents BOI-T.





**Figure S7** XPS spectra for I 3d of BOI-T and BOI-P, green line represents BOI-P, blue line represents BOI-T.





**Figure S8** Products yield without catalyst.





**Figure S9** Products yield under Ar atmosphere of BOI-P and BOI-T. Green line represents BOI-P and blue line represents BOI-T.


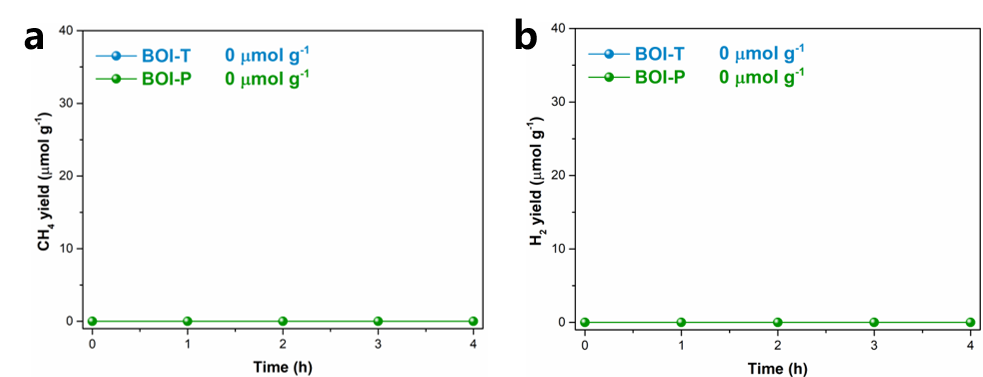


**Figure S10** CH_4_ (**a**) and H_2_ (**b**) yield of BOI-P and BOI-T under simulated solar light. Green line represents BOI-P and blue line represents BOI-T.


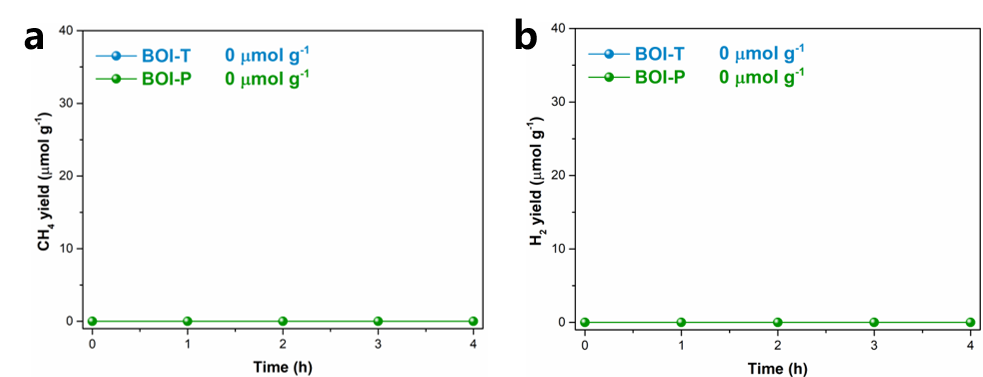


**Figure S11** CH_4_ (**a**) and H_2_ (**b**) yield of BOI-P and BOI-T under visible light (λ > 420 nm). Green line represents BOI-P and blue line represents BOI-T.


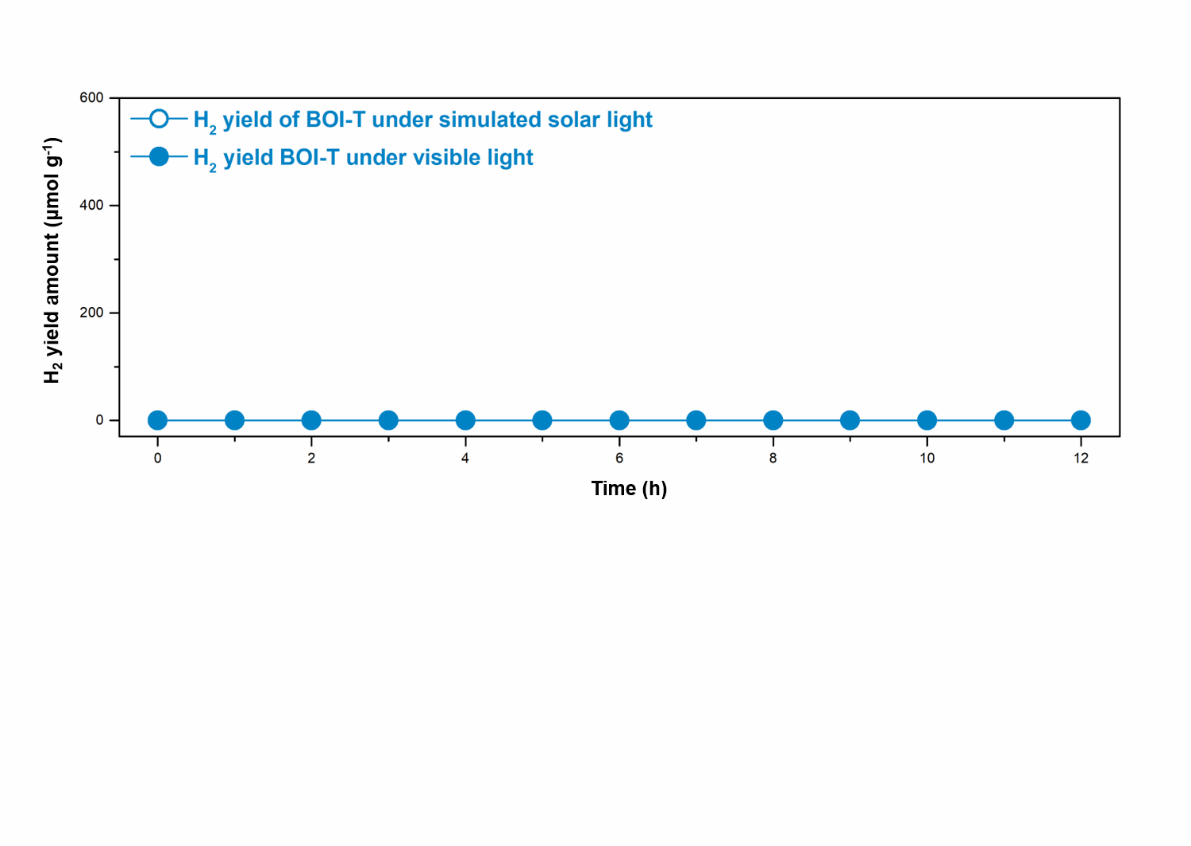


**Figure S12** H_2_ yield of BOI-T after 12 h CO_2_ photoreduction.


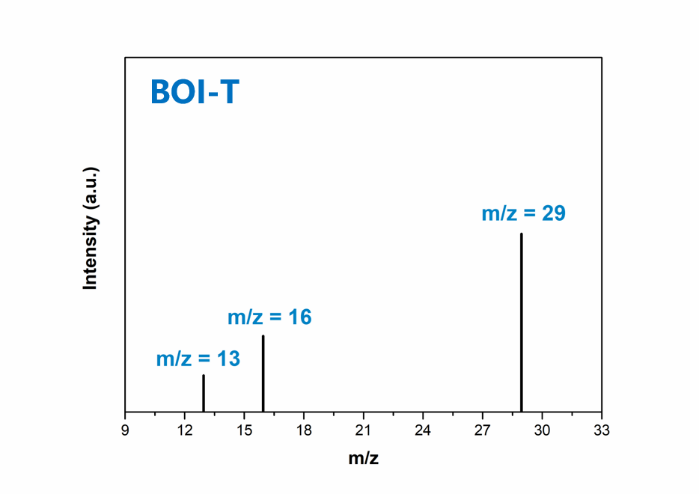


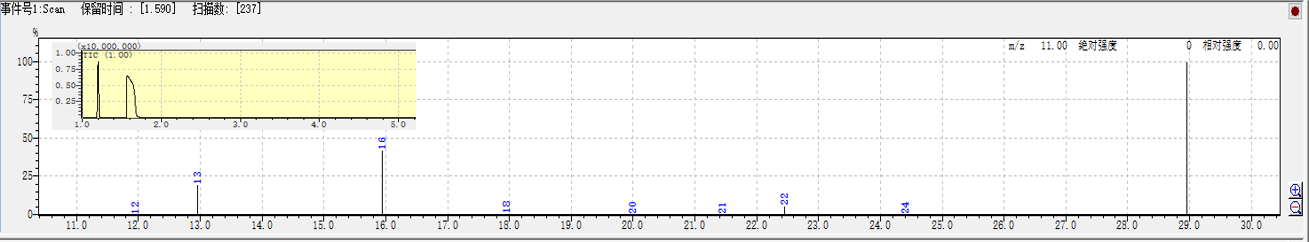


**Figure S13** ^13^CO_2_ isotopic labeling experiments with BOI-T.





**Figure S14** EPR signals for electron trapping of BOI-P and BOI-T. Black line represents balnk, green line represents BOI-P and blue line represents BOI-T.





**Figure S15** BET of BOI-P and BOI-T. Green line represents BOI-P and blue line represents BOI-T.


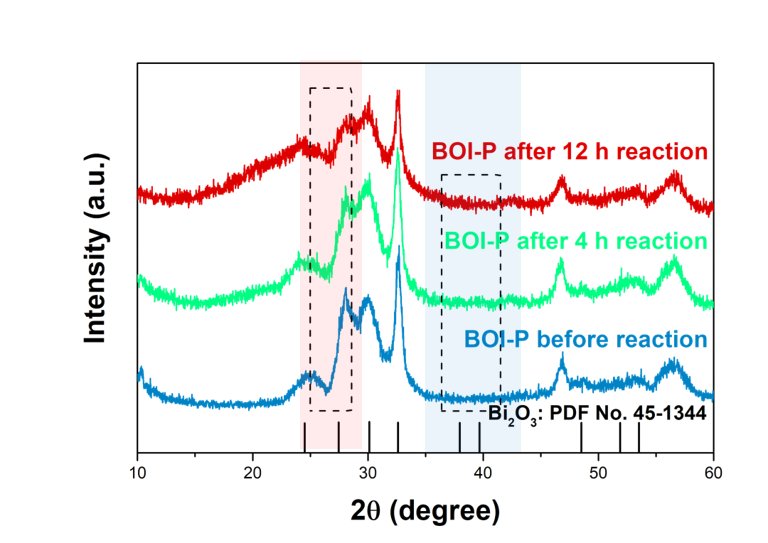


**Figure S16** XRD of BOI-P before and after 4 h, 12 h CO_2_ photoreduction under visible light illumination. Blue line represents BOI-P before reaction, green line represents BOI-P after 4 h reaction, and red line represents BOI-P after 12 h reaction.


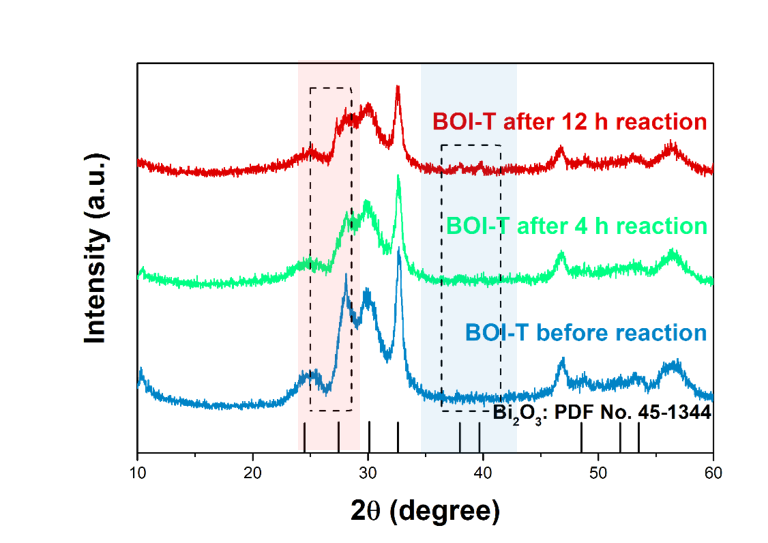


**Figure S17** XRD of BOI-T before and after 4 h, 12 h CO_2_ photoreduction under visible light illumination. Blue line represents BOI-T before reaction, green line represents BOI-T after 4 h reaction, and red line represents BOI-T after 12 h reaction.


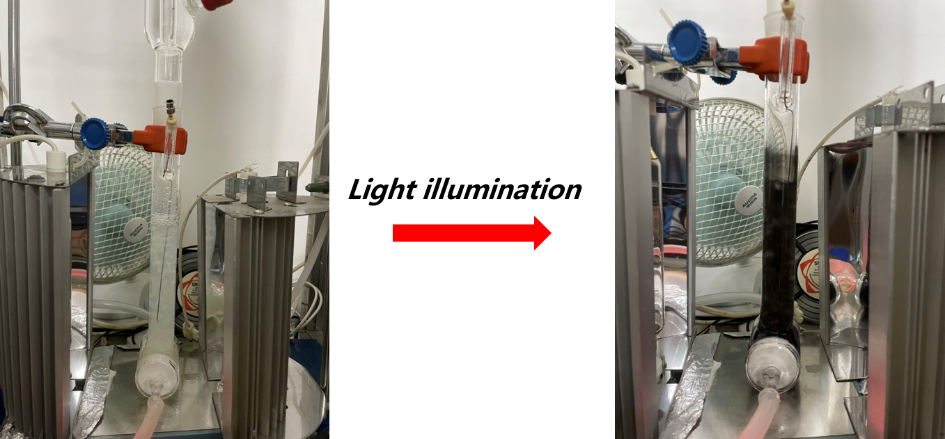


**Figure S18** The color change of BOI-T under visible light illumination.


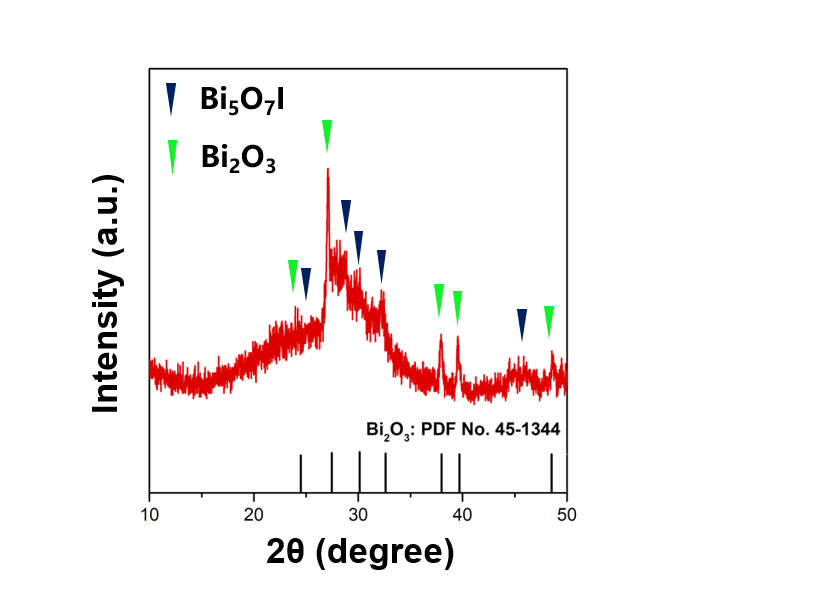


**Figure S19** XRD pattern of BOI-T after 20 min of visible light illumination.





**Figure S20** XRD pattern of recovered BOI-T.


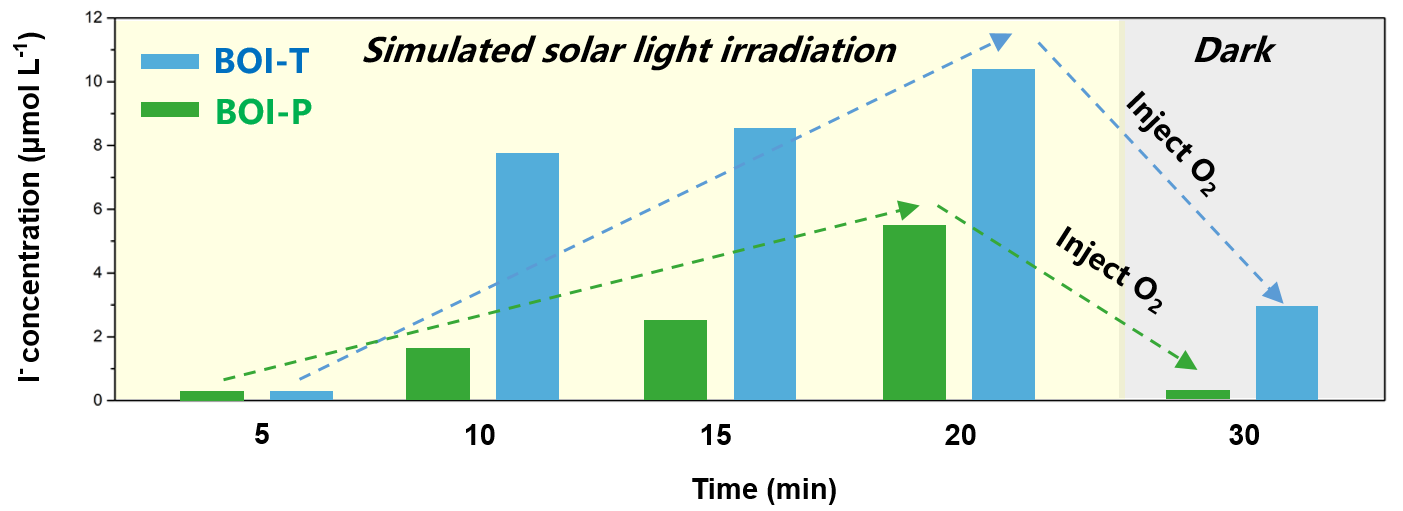


**Figure S21** Variation trend of free I^-^ in solution under simulated solar light illumination detected by ICP.





**Figure S22** EPR signals for defect detection of BOI-P and BOI-T under simulated solar light. Green line represents BOI-P and blue line represents BOI-T.


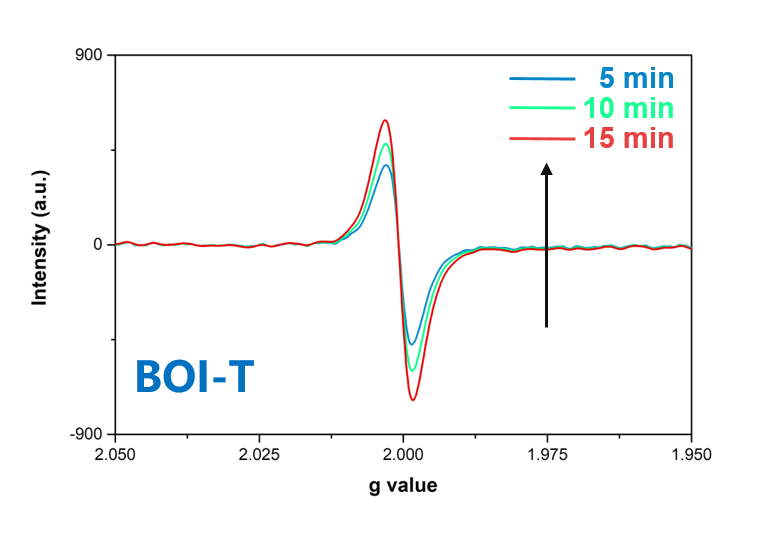


**Figure S23** EPR signals for defect detection of BOI-T under continuous simulated solar light illumination.


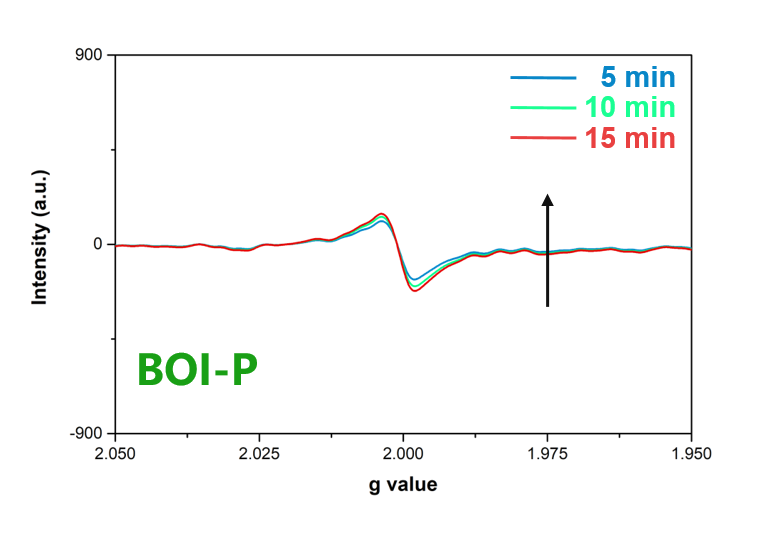


**Figure S24** EPR signals for defect detection of BOI-P under continuous simulated solar light illumination.


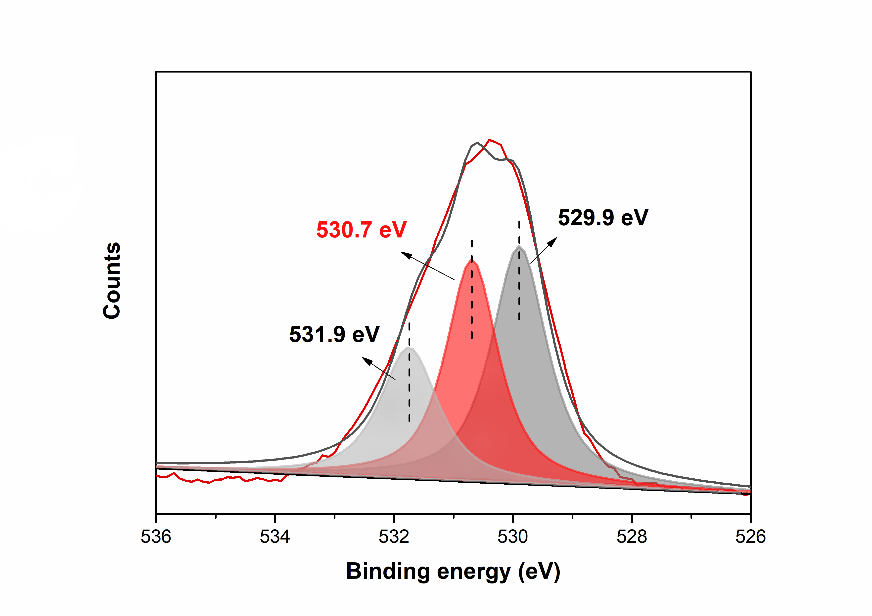


**Figure S25** XPS spectra for O 1s of discolored BOI-T.





**Figure S26** EPR signal for defect detection of discoloured BOI-T after visible light illumination treatment.





**Figure S27** EPR signals for electron trapping of BOI-T and BOI-T with I defects under visible light illumination. Black line represents blank, blue line represents BOI-T and red line represents BOI-T with I defects.





**Figure S28** UV-vis DRS of BOI-T and BOI-T with I defects. Blue line represents BOI-T and red line represents BOI-T with I defects.


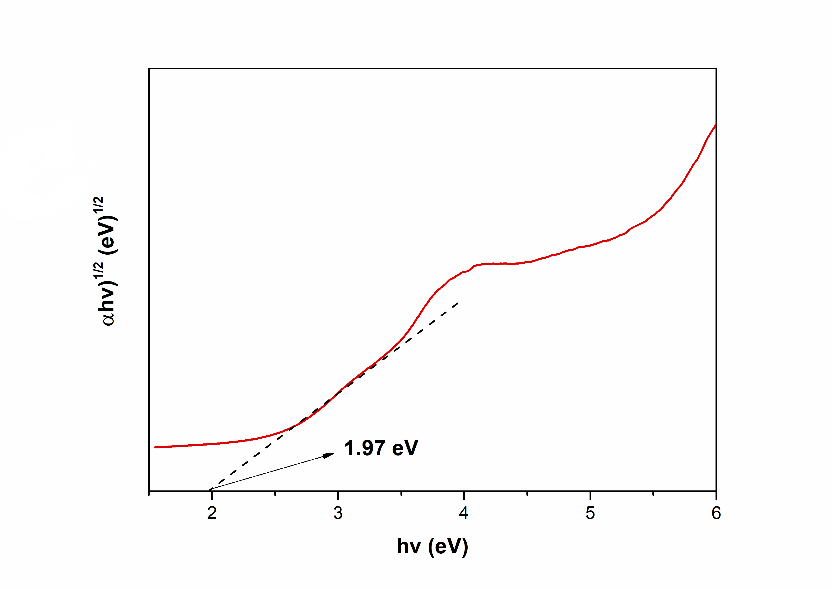


**Figure S29** Tauc plot of BOI-T with I defects.





**Figure S30** In situ FT-IR spectra of the CO_2_ photoreduction process over BOI-T recorded during simulated solar light irradiation cycles of 1, 4, 10, 20, 40, 80, 120, 140, 160, and 180 (from bottom to top).





**Figure S31** In situ FT-IR spectra of the CO_2_ photoreduction process over BOI-P recorded during simulated solar light irradiation cycles of 1, 4, 10, 20, 40, 80, 120, 140, 160, and 180 (from bottom to top).


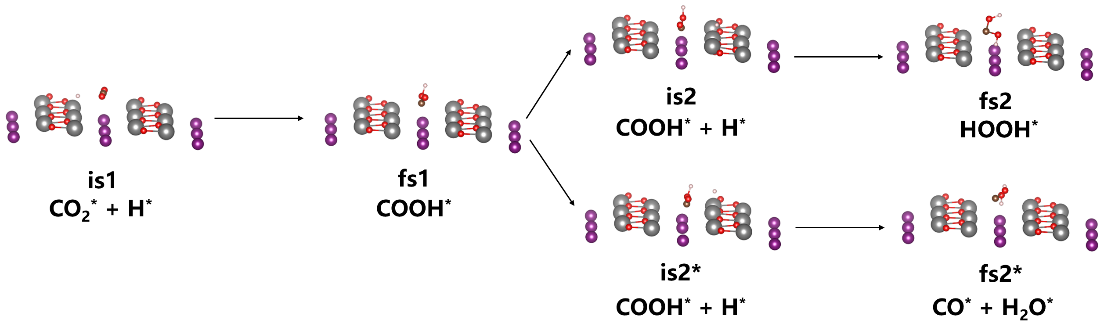


**Figure S32** Model structures of each step of CO_2_ photoreduction of BOI-pri.


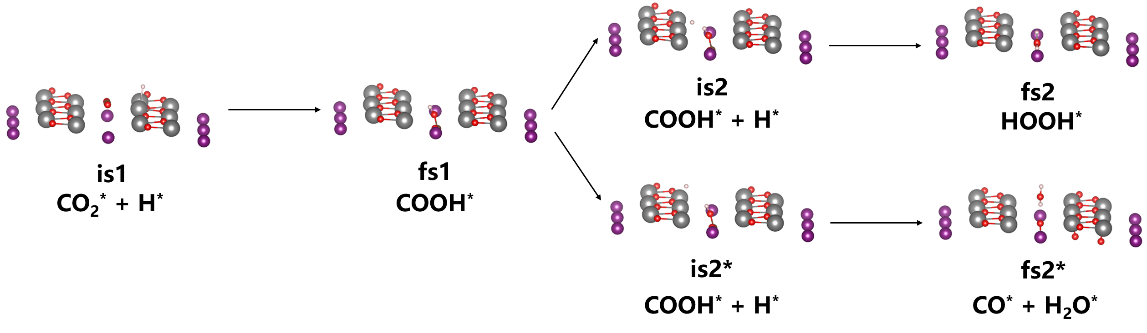


**Figure S33** Model structures of each step of CO_2_ photoreduction of BOI-pri-I.


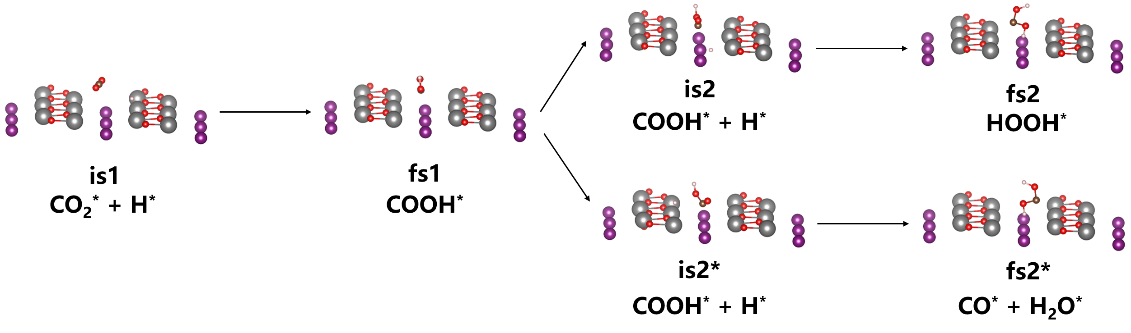


**Figure S34** Model structures of each step of CO_2_ photoreduction of BOI-strain.


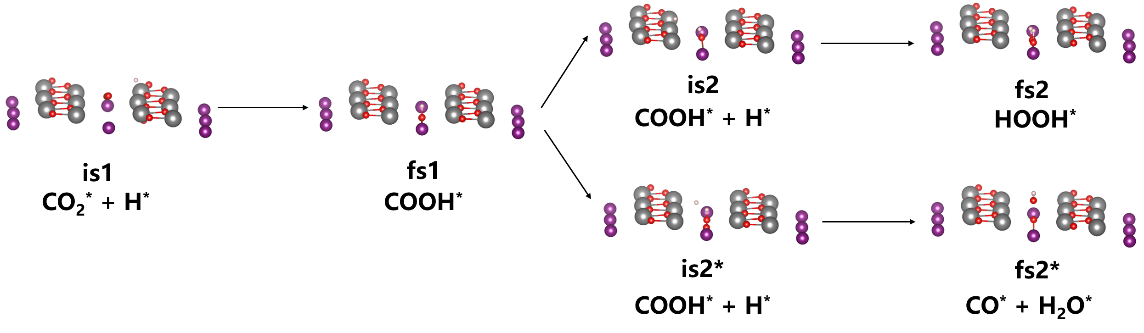


**Figure S35** Model structures of each step of CO_2_ photoreduction of BOI-strain-I.

**Supplementary Tables**

**Supplementary Table S1. Comparison of the CO_2_ photoreduction activity to yield CO with other catalysts.**

| Entry | Photocatalyst | Light source | CO production  (μmol g^-1^ h^-1^) | Product selectivity  (%) | Ref |
| --- | --- | --- | --- | --- | --- |
| 1 | Bi_3_O_4_Cl/20%g-C_3_N_4_ | 300W Xe lamp | 6.6 | 46.5% | 1 |
| 2 | hollow Bi_4_O_5_Br_2_ | 300W Xe lamp | 3.16 | 61.2% | 2 |
| 3 | 5%Bi_2_Se_3_/g-C_3_N_4_ | 300W Xe lamp | 8.2 | —— | 3 |
| 4 | Atomically-thin Bi_2_MoO_6_ | 300W Xe lamp | 3.62 | —— | 4 |
| 5 | Bi_4_O_5_BrI | 300W Xe lamp | 22.85 | 72.8% | 5 |
| 6 | BiOCl | 500 W Xe lamp | 1.01 | 62.7% | 6 |
| 7 | BiOBr | 300 W Xe lamp ( λ > 400 nm) | 1.68 | 72.4% | 7 |
| 8 | BiOI | 300 W Xe lamp (λ > 400 nm) | 0.51 | 41.5% | 8 |
| 9 | Bi_5_O_7_I | 300 W Xe lamp (λ > 400 nm) | 1.73 | 70.6% | 9 |
| 10 | Cu-g-C_3_N_4_ | 300W Xe lamp | 3.09 | Nearly 100% | 10 |
| 11 | g-C_3_N_4_/BiOI | 300 W Xe lamp (λ > 400 nm) | 4.86 | —— | 11 |
| 12 | Bi_12_O_17_Br_2_ nanotubes | 300W Xe lamp | 28.30 | —— | 12 |
| 13 | Oxygen-vacancies  rich Sr_2_Bi_2_Nb_2_TiO_12_ | 300 W Xe  lamp | 17.11 | —— | 13 |
| 14 | BiOIO_3_ | 300 W Xe  lamp | 17.33 | —— | 14 |
| 15 | Oxygen-vacancies  rich WO_3_ layers | IR light  (silicon nitride  lamp) | 2.80 | —— | 15 |
| 16 | InVO_4_ nanosheets | 300 W Xe  lamp | 18.28 | —— | 16 |
| 17 | α-Fe_2_O_3_/g-C_3_N_4_ | 300 W Xe  lamp | 27.20 | —— | 17 |
| **18** | **Bi_5_O_7_I with I defects** | **300 W Xe**  **Lamp (AM 1.5G)** | **65.10** | **100%** | **This work** |
| **19** | **Bi_5_O_7_I with I defects** | **Visible light**  **(λ > 420 nm)** | **15.45** | **100%** | **This work** |

**Supplementary supporting references:**

[1] Y. Xu, X. Jin and T. Ge et al., “Realizing Efficient CO_2_ Photoreduction in Bi_3_O_4_Cl: Constructing Van der Waals Heterostructure with g-C_3_N_4_,” *Chemical Engineering Journal*, vol. 409, pp. 128178-128186, 2021.

[2] X. Jin, C. Lv and X. Zhou et al., “A bismuth rich hollow Bi_4_O_5_Br_2_ photocatalyst enables dramatic CO_2_ reduction activity,” *Nano Energy*, vol. 64, pp. 103955-103962, 2019.

[3] Y. Huang, K. Wang, T. Guo, J. Li, X. Wu and G. Zhang, “Construction of 2D/2D Bi_2_Se_3_/g-C_3_N_4_ nanocomposite with High interfacial charge separation and photo-heat conversion efficiency for selective photocatalytic CO_2_ reduction,” *Applied Catalysis B Environmental*, vol. 277, p. 119232, 2020.

[4] J. Di, X. Zhao and C. Lian et al., “Atomically-thin Bi_2_MoO_6_ nanosheets with vacancy pairs for improved photocatalytic CO_2_ reduction,” *Nano Energy*, vol. 61 pp. 54-59, 2019.

[5] Y. Bai, L. Ye and T. Chen et al., “Synthesis of hierarchical bismuth-rich Bi_4_O_5_Br_x_I_2-x_ solid solutions for enhanced photocatalytic activities of CO_2_ conversion and Cr(VI) reduction under visible light,” *Applied Catalysis B Environmental*, vol. *203*, pp. 633-640, 2017.

[6] L. Zhang, W. Wang, D. Jiang, E. Gao and S. Sun, “Photoreduction of CO_2_ on BiOCl nanoplates with the assistance of photoinduced oxygen vacancies,” *Nano Research*, vol. 8, pp. 821-831, 2015.

[7] L. Ye, X. Jin and C. He et al., “Thickness-ultrathin and bismuth-rich strategies for BiOBr to enhance photoreduction of CO_2_ into solar fuels,” *Applied Catalysis B Environmental*, vol. 187, pp. 281-290, 2016.

[8] L. Ye, H. Wang and X. Jin et al., “Synthesis of olive-green few-layered BiOI for efficient photoreduction of CO_2_ into solar fuels under visible/near-infrared light,” *Solar Energy Materials & Solar Cells*, vol. 144, 732-739, 2016.

[9] C. Ding, L. Ye and Q. Zhao et al., “Synthesis of Bi_x_O_y_I_z_ from molecular precursor and selective photoreduction of CO_2_ into CO,” [*Journal of CO_2_ Utilization*](http://www.baidu.com/link?url=a9quHdwEq_eOdHRkJWIFC9V_nM_gL1c8dFWvAmDGsWT8X8ZDvq989tjhecOXSA6FpWxi_gasWIeR5PoEowzRLq), vol. 14, 135-142, 2016.

[10] Y. Li, B. Li, D. Zhang, L. Cheng and Q. Xiang, “Crystalline Carbon Nitride Supported Copper Single Atoms for Photocatalytic CO_2_ Reduction with Nearly 100% CO Selectivity,” *ACS Nano*, vol. 14, no. 8, pp. 10552-10561, 2020.

[11] J. Wang, H. Yao, and Z. Fan et al., “Indirect Z-Scheme BiOI/g-C_3_N_4_ Photocatalysts with Enhanced Photoreduction CO_2_ Activity under Visible Light Irradiation,” *ACS Applied Materials & Interfaces*, vol. 8, no. 6, pp. 3765-3775, 2016.

[12] J. Di, P. Song and C. Zhu et al., “Strain-engineering of Bi_12_O_17_Br_2_ nanotubes for boosting photocatalytic CO_2_ reduction,” *ACS Materials Letters*, vol. 2, no. 8, pp. 1025-1032, 2020.

[13] H. Yu, J. Li and Y. Zhang et al., “Three-in-One Oxygen Vacancies: Whole Visible-Spectrum Absorption, Efficient Charge Separation, and Surface Site Activation for Robust CO_2_ Photoreduction,” *Angewandte Chemie International Edition,* vol. 58, no. 12, pp. 3880-3884, 2019.

[14] F. Chen, H. Huang, L. Ye, T. Zhang and Y. Zhang, “Thickness‐dependent facet junction control of layered BiOIO_3_ single crystals for highly efficient CO_2_ photoreduction," *Advanced Functional Materials*, vol. 28, no. 46, p. 1804284, 2018.

[15] L. Liang, X. Li and Y. Sun et al. “Infrared Light-Driven CO_2_ Overall Splitting at Room Temperature,” *Joule*, vol. 2, no. 5, pp. 1004-1016, 2018.

[16] Q. Han, X. Bai and Z. Man et al., “Convincing Synthesis of Atomically Thin, Single-Crystalline InVO_4_ Sheets toward Promoting Highly Selective and Efficient Solar Conversion of CO_2_ into CO,” *Journal of American Chemical Society*, vol. 141, no. 10, pp. 4209-4213, 2019.

[17] Z. Jiang, W. Wan, H. Li, S. Yuan, H. Zhao and P. Wong, “A Hierarchical Z-Scheme α-Fe_2_O_3_/g-C_3_N_4_ Hybrid for Enhanced Photocatalytic CO_2_ Reduction,” *Advanced Materials*, vol. 30, no. 10, p. 1706108, 2018.

**Supplementary Table S2.** Comparison of the stability of CO_2_ photoreduction of BOI-T with other catalysts

| Catalyst | Light source | Reaction medium | CO Selectivity | CO generation rate (μmol g^-1^ h^-1^) | Stability (h) | Ref. |
| --- | --- | --- | --- | --- | --- | --- |
| **Bi_5_O_7_I with I defects** | **Visible light (λ > 420 nm)** | **Gas-solid, water** | **100%** | **15.45** | **12** | **This work** |
| g-C_3_N_4_/NiAI-LDH | Xe lemp | Gas-solid, water | 82% | 8.2 | 20 | 1 |
| α-Fe_2_O_3_/Cu_2_O | Xe lemp | Gas-solid, water | ~100% | 1.67 | 3 | 2 |
| Cd_1-x_Zn_x_S | LED light | Gas-solid, water | 95% | 2.9 | 5 | 3 |
| Cu-I-TiO_2_ | Xe lemp | Gas-solid, water | ~100% | 6.7 | 3.5 | 4 |
| PB-Bi_2_WO_6_ | Xe lemp | Gas-solid, water | ~100% | 0.33 | 18 | 5 |
| ZnO_1-x_/C | Xe lemp | Gas-solid, water | ~100% | ~18.19 | 15 | 6 |

**Supplementary supporting references:**

[1] S. Tonda, S. Kumar, M. Bhardwaj, P. Yadav and S. Ogale, “g-C_3_N_4_/ NiAl-LDH 2D/2D Hybrid Heterojunction for High-Performance Photocatalytic Reduction of CO_2_ into Renewable Fuels,” *ACS Applied Materials & Interfaces*, vol. 10, no. 3, pp. 2667-2678, 2018.

[2] J. Wang, L. Zhang and W. Fang et al., “Enhanced Photoreduction CO Activity over Direct Z-Scheme α-FeO/CuO Heterostructures under Visible Light Irradiation,” *ACS Applied Materials & Interfaces*, vol. 7, no. 16, pp. 8631-8639, 2015.

[3] E. A. Kozlova, M. N. Lyulyukin, D. V. Markovskaya, D. S. Selishchev, S. V. Cherepanova and D. V. Kozlov, “Synthesis of Cd_1-x_Zn_x_S Photocatalysts for Gas-phase CO_2_ Reduction under Visible Light,” *Photochemical & Photobiological Sciences*, vol. 18, pp. 871-877, 2019.

[4] Q. Zhang, T. Gao, J. M. Andino and Y. Li, “Copper and Iodine Co-modified TiO_2_ Nanoparticles for Improved Activity of CO_2_ Photoreduction with Water Vapor,” *Applied Catalysis B Environmental*, vol. 123, pp. 257-264, 2012.

[5] Z. Sun, Z. Yang, H. Liu, H. Wang and Z. Wu, “Visible-light CO_2_ Photocatalytic Reduction Performance of Ball-flower-like Bi_2_WO_6_ Synthesized without Organic Precursor: Effect of Post-calcination and Water vapor,” *Applied Surface Science*, vol. 315, pp. 360-367, 2014.

(6) L. Lin, S. Kavadiya and B. Karakocak et al., “ZnO_1-x_/carbondots Composite Hollow Spheres: Facile Aerosol Synthesis and Superior CO_2_ Photoreduction under UV, Visible and Near-infrared irradiation,” *Applied Catalysis B Environmental*, vol.230, pp. 36-48, 2018.
